# Abstract from the Minutes of Atlanta Medical Society

**Published:** 1861-04

**Authors:** 


Vol. VI.] APRIL, 1861. [No. 8.

### ORIGINAL COMMUNICATIONS.

### ARTICLE I.

### *Abstract from the Minutes of Atlanta Medical Society.*

Atlanta Medical Society, Jan. 11, 1861.

There being no cases to report, the subject for discussion was declared in order by the President, viz: "The contagiousness of Typhoid fever."

*Dr. J. P. Logan* was of the opinion the fever is communicable. He thought it might be transmitted from one person to another, from his own observation on this subject. Why may it not be contagious or communicable? The disease is a special one, dependent upon a special virus, and therefore may, it strikes me, be communicated. I have seen cases which seemed to be traceable to contagious origin, and from the reports of physicians, on this subject, we have the evidence of eminent men in the profession going to sustain this position.

*Dr. H. Coe* thought typhoid fever not contagious in a pure atmosphere, and those cases which seem to be taken from exposure to the disease, are most likely contracted from the atmosphere. The atmosphere may be rendered less healthy in the immediate presence of the subject of this fever, and persons breathing it may be more liable to contract the disease under such circumstances than if no such additional influence existed, but would not be taken unless the epidemic poison existed in the atmosphere independent of the idio-miasmata. Persons may be more subject under these combined influences, but from confinement in a room with a case of the disease in a section of country where no disposition in the air existed, it would not be contracted. Does not know what is the peculiarity of the atmosphere that produces the fever, further than that it is probably one of those inexplicable states called epidemic. Has no treatment for the disease, farther than the means necessary to support the system, and to prevent the ravages of the various forms of local disturbance so common in this affection. Believes the seat of the disease to be in the nervous system, probably the ganglionic system of nerves, particularly.

*Dr. J. G. Westmoreland* endorsed the opinion of Dr. Coe, so far as the non-contagiousness of this fever.

During ten years' experience in the treatment of the disease no instance of its being communicated from one person to another, has been satisfactorily established—constant confinement in the room with the sick, even on the bed with them, will not, so far as he had witnessed, cause it to be contracted. In answer to Dr. Logan's question "why may it not be communicated?" he said, "*why may it be communicated?*" The of that whole neighborhoods being taken one after another, and many at the same time, are not sufficient reasons for believing that it is communicated from one to another; because, other diseases, admitted by all to be non-contagious, pursue precisely the same course in this particular. Influenza, diphtheria and pneumonia are often epidemic, and give the same reasons for suspecting contagion as typhoid fever; nor do we know any more of the peculiar electrical or atmospheric changes necessary to their origin than in the production of fever. Each disease just mentioned, requires for its development a particular condition of the atmosphere, differing from those which cause the others, and each affects particular organs, and in a particular way. Those most susceptible, from particular states of the system, favoring the development, are first attacked, when exposed to any particular epidemic.

*Dr. D. C. O'Keefe*—Why is it that physicians advise the well not to visit those affected with this fever? Believes it contagious, but more so in certain localities, and under particular circumstances, than otherwise.

*Dr. H. L. Wilson* rose merely to say, that inasmuch as it had been contended by members of the profession, that the analogy which seems to exist between typhoid fever and diseases known to be contagious, in the immunity against a second attack, was an argument in favor of its contagiousness, he wished to give some facts, as they had been reported to him, going to prove that persons are not exempt from a second attack of typhoid fever. He instanced two cases reported to him by the attending physician.

*Dr. H. W. Brown*—Had read Bartlett on typhoid fever until the subject became so mistified that he never yet formed any definite conclusion in regard to the subject under discussion. He had been impressed, however, with the belief of its contagiousness, from the result of close observations in more than one instance of this fever being contracted under circumstances favoring its contagious character.

JANUARY 18, 1861.

The report of cases being in order,

*Dr. Brown* mentioned a case of enteritis which had recently come under his observation from indulgence in indigestible food. He reported it in order to call the attention of members to the fact that care is necessary in the selection of digestible articles of diet, for patients whose organs of digestion have been enfeebled by protracted and violent disease. The case was that of a child convalescing from typhoid fever.

*Dr. Denny* reported, *in writing*, a case of delirium tre mens, and called the attention of the society to what he considered points of interest, viz: the relapse of the patient during the operation of oleum ricini, after all the symptoms of delirium had passed away, and the fact that the habit of intemperance had been commenced and continued under the use of medicinal alcohol, instead of the usual liquors imbibed by the devotees of bacchus.

The subject for discussion, "Best procedure in placenta-præuia," being in order,

*Dr. Brown* mentioned two instances in which he found it necessary to detach the placenta. He alluded to turning, but does not approve of this mode of delivery in all cases where it is possible to resort to it. The head of the child acting as a tampon, after the placenta is removed, effectually arrests the hemorrhage, so that no haste is required on that score.

The necessity for turning, however, can be determined in each particular case, and when necessary should be resorted to.

*Dr. J. G. Westmoreland* desired to call the attention of members to the importance of adopting such means as will save the life of both mother and child, if possible. By separating the placenta at once, we, of course, sacrifice the child, which should not be done, unless in doing so the only hope of saving the mother is found. He favored turning at once as the best mode of effecting these objects, when the hemorrhage is sufficiently free to endanger the life of the mother, provided the os uteri be in condition to allow it—does not approve of waiting when the symptoms justify action.

*Dr. Denny* advocated speedy delivery as the best mode of preserving the lives of the mother and child, and contended that turning was the best proceedure when it could be effected. He alluded to the danger of an attempt at turning, when the os uteri is not in a condition suitable for such operation.

